# Imaging the spatiotemporal dynamics of covert attention with a pupillometric probing paradigm

**DOI:** 10.1016/j.isci.2026.115386

**Published:** 2026-03-18

**Authors:** Marnix Naber, Marinos Savva, Lotte van den Berg, Stefan Van der Stigchel, Samson Chota

**Affiliations:** 1 Experimental Psychology, Helmholtz Institute, Faculty of Social and Behavioral Sciences, Utrecht University, 3584CS Utrecht, the Netherlands

**Keywords:** Cognitive neuroscience, Neuroscience, Techniques in neuroscience

## Abstract

We can use a covert form of selective attention to efficiently inspect visual aspects in the periphery without making eye movements. However, it has been a challenge to reveal how covert attention shifts across time and space. Here, we report on a pupillometric orienting method to directly probe and visualize covert shifts of attention between cues and targets. The analysis of the spatiotemporal dynamics of attention-enhanced pupil responsivity to probes, presented around and between cues and targets, indicates that attention shifts across spatial locations between cues and targets despite the absence of interconnected objects guiding attention. Furthermore, current settings allowed to make rough temporal estimates on how long it takes for attention to start moving and for it to arrive at target locations. These findings lay the basis for future research into more detailed estimates of departure and arrival latencies, which may vary depending on shift directions, stimulus designs, and populations.

## Highlights

• A pupillometric probing method images covert attention across space and time
• Covert attention shifts as a moving spotlight between cue and target locations
• Attention passes through intermediate locations without object-based guidance
• The method enables rough estimates of attentional onset, speed, and arrival latencies

## Graphical abstract

**Figure:**
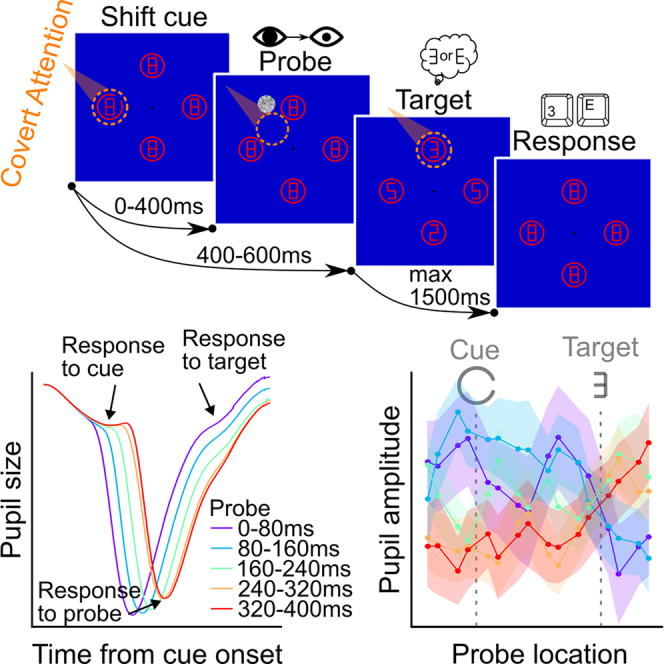

## Introduction

**Figure 1:**
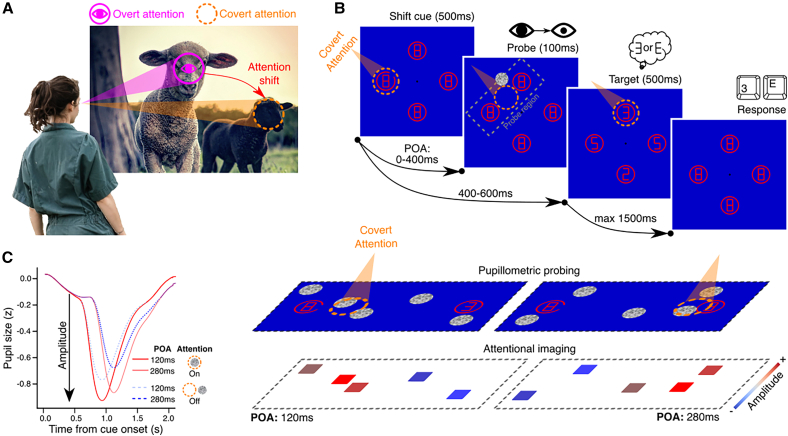
Attention types, procedure, and paradigm (A) Observers allocate covert attention (orange cone) to a region in the visual periphery, for example, to determine where to shift overt attention (magenta) next. Farmer photo by Zoe Schaeffer (unsplash.com); Sheep photo by COCO image dataset (cocodataset.org). (B) Experimental cueing paradigm with a circle changing to an arc, cueing a direction for where to shift attention to. Digital eighths change to either non-targets (2’s and 5’s) or targets (3’s or E’s) for the identification task. To evoke a pupil response per trial, one probe was presented after a random interval after cue onset (i.e., probe-onset-asynchrony; POA) and at a position within a rectangular region around cue and target (dotted gray; not shown to observers). Observers maintain fixation at the screen’s center (black dot) while covertly shifting attention (represented as a red spotlight; not shown during the experiment) between cues and targets in (counter)clockwise directions. (C) Attentional imaging paradigm for which probes evoked a pupil response, with strong versus weak constriction amplitudes, respectively, indicating more (red) versus less (blue) attention allocated to the probe’s location at the time of appearance. One probe was presented per trial, but the panel shows multiple probes to clarify how these accumulate into an imaged attention map.

Our visual environment is incredibly rich, both in relevant and irrelevant information. While our eyes’ retina can capture large parts of visual scenes, only its central part, the fovea, provides high acuity information about fixated objects. Eye movements produce *overt* (i.e., visible behavior) shifts of attention by guiding the fovea through potentially relevant locations in the visual environment in a sequential manner. However, *covert* attention (i.e., a not directly observable operation), occurring in the absence of visible eye movements, allows us to selectively enhance processing of objects both within and outside of the fovea.^1^^,^^2^^,^^3^^,^^4^ Covert attentional processing can be allocated independently of the location of gaze^5^^,^^6^: One may stare at an object, but attend another location in the periphery (Figure 1A). This form of covert attention is especially useful for a variety of reasons. First, it allows for efficient inspection of the periphery without making costly eye movements.^7^^,^^8^^,^^9^ Second, covert attention enables the selection and preparation of visual processing of peripheral objects before overt inspection.^10^^,^^11^^,^^12^^,^^13^^,^^14^ Last, pre-saccadic shifts of covert attention may stabilize perception despite the frequent, abrupt visual changes on the fovea caused by rapid eye movements.^7^^,^^8^^,^^15^ Although numerous discoveries highlight the functional significance of covert shifts of attention, a fundamental question has remained unanswered: How exactly does a shift of attention from one location to another unfold in time and space conjunctively? More specifically, what are its spatiotemporal dynamics?

Over the years, studies tried to measure either a spatial *or* temporal component of attention, relying on conventional trial-by-trial behavioral reports (e.g., reaction times) or neural measures e.g.,.^16^^,^^17^ Such studies have led to valuable insights into the characteristics and mechanisms of attention (for reviews, see,^18^^,^^19^) such as the current view that covert attention is a “mental spotlight,”^20^^,^^21^^,^^22^ and that covert attention shifts within 100-300 ms.^23^^,^^24^^,^^25^^,^^26^^,^^27^^,^^28^^,^^29^^,^^30^^,^^31^^,^^32^ Some studies have aimed to investigate how covert attention shifts across space *and* time, but the literature has not yet settled on how to best describe the spatiotemporal dynamics. Evidence from both behavioral and electrophysiological work suggests that attention sticks to a start point and at the same time stretches out to cover an object (or space) over time,^33^^,^^34^^,^^35^^,^^36^^,^^37^ also referred to as a *spread* of attention. Other behavioral studies suggest that the spatial form or distribution of attention does not change, but changes position entirely as a local spotlight or beam from location to location.^38^^,^^39^^,^^40^^,^^41^ This literature suggests that attention disappears from the start point, and this is referred to as a *move*. As it remains unclear which of these dynamics best describe how attention behaves (but see discussion), we will continue to use the umbrella term *shift*. Nonetheless, it is important to note that the spreading of attention may rely on the presence of interconnected and collinear lines or objects that guide the path of attention.^42^^,^^43^ Thus, although it remains unclear whether peripheral facilitations by attention are caused by an object-based spread or by a locally moving spotlight, the distribution of attentional resources seems to shift across space and facilitates the processing of locations between fixation and peripheral targets.

To learn how attention shifts across locations, it is necessary to measure attention across a broad spatial and temporal span rather than at a limited number of locations or time points, as tested in previous studies. Only a few studies have managed to create maps of the spatial distribution of attention using sophisticated neuroscientific methods (fMRI, MEG, and EEG), but the resulting maps either lack a temporal component^44^^,^^45^^,^^46^ or are of too low spatial resolution to draw conclusions about their spatiotemporal dynamics.^47^ This asks for an alternative approach.

Here, we aim to demonstrate with the use of a pupillometric probing paradigm that pupil responses to stimuli allow tracking changes in attention across *both* space and time. This at a resolution high enough to create images of the space between a start and target location for the time points between cue onset, the take-off of attention, and its arrival at the target. Due to the pupil size’s sensitivity to attentional modulations,^48^^,^^49^^,^^50^^,^^51^^,^^52^^,^^53^^,^^54^^,^^55^ we can limit the number of trials per spatial and temporal segment. In three experiments, observers were tasked with covertly shifting attention between locations to accurately identify a cued target. During these attentional shifts, we present a task-irrelevant visual probe at varying time points and spatial locations. We hypothesize that these probes would lead to differential changes in pupil size depending on their spatial and temporal overlap with the current focus of attention. Visualizing pupil response amplitudes in multiple two-dimensional snapshots at sequential time points allows us to characterize how the attentional spotlight shifts from one location to another.

## Results

### Experiment 1 – Covert shifts within the periphery

The procedure that allows for probing changes in attention across visual space and time consists of the combination of an attentional cueing task and pupillometric probe paradigm (Figure 1B). The attentional cueing paradigm follows a standard protocol during which observers covertly direct attention from a start position (cue location) toward a distinct target location to enhance target identification.^3^ Observers are instructed to fixate the center of a screen at all times while four placeholders (digital eights embedded in circles) are continuously presented in the para-fovea (8 degrees of visual angle; dva) at the cardinal axis of the visual field. The placeholder’s circles serve as cues; by opening a portion of the circle, observers are cued toward where to allocate their covert attention next (clockwise or counter-clockwise directions). Second, the digital eights inside the placeholders serve as potential targets; around 400-600 ms after the cue, the cued target briefly changes to either a “3” or an “E,” while the stimuli at all other locations change into non-targets (“2s” or “5s”).

The pupillometric probe paradigm enables the measurement of the distribution of attentional resources across time and space between cues and targets (Figure 1C). More specifically, the amplitude of convoluted pupillary responses to sudden probe onsets, consisting of a transient constriction, reflects the degree of attention allocated at the location and moment of the probe’s appearance. Importantly, the constriction amplitude scales with the amount of attentional resources allocated to the probed location around its onset.^48^^,^^51^ The distribution of attentional resources is thus measured by the proxy of pupil response amplitudes.

**Figure 2:**
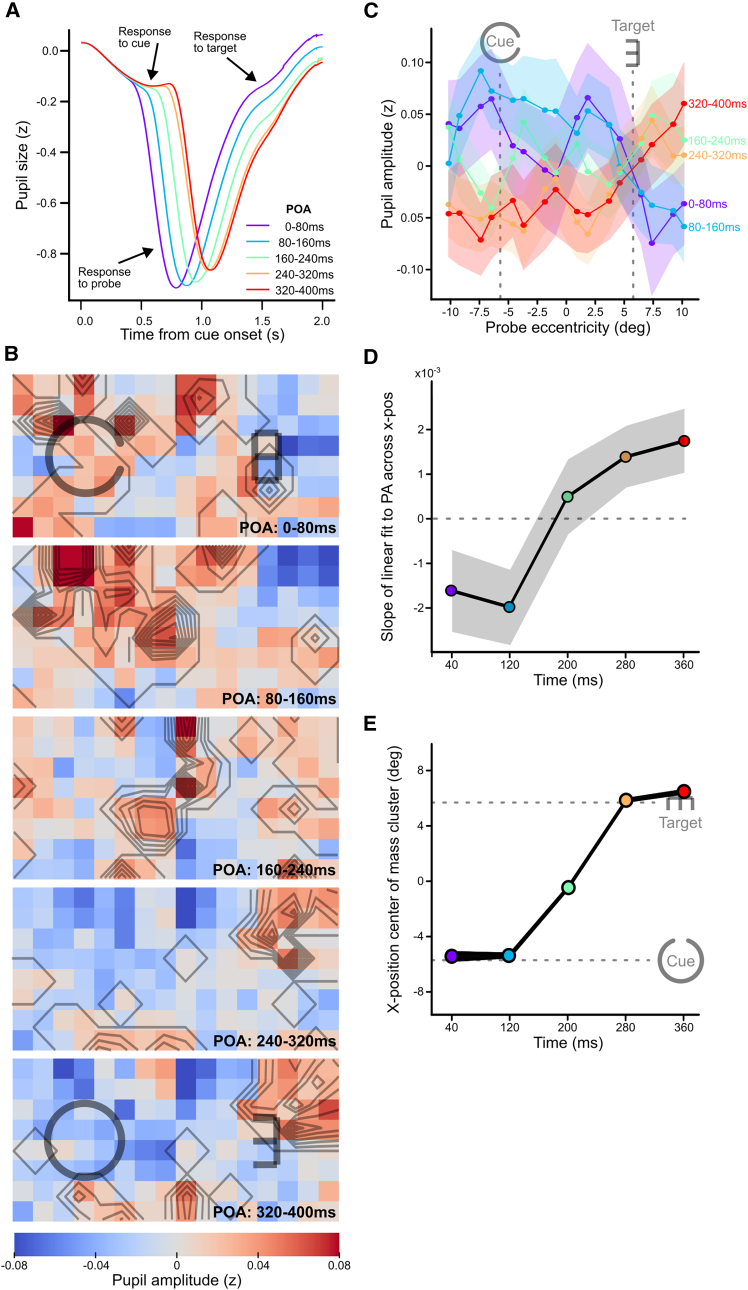
Pupil responses and imaged maps of covert shifts of attention between peripheral locations (A) Pupil responses to probe onsets per POA interval (colors) averaged across trials and observers. Increased latencies for longer POAs confirm that probe onsets contributed most strongly to the pupil responses, with only minor effects of cue and target onsets (see arrows). The amplitude of the larger constrictions was expected to increase for probes presented inside the focus of covert attention. (B) Each panel shows the baseline-corrected attentional imaging result (for details, see Figure S1) of a selective POA interval to visualize pupil amplitude modulations caused by the shift of covert attention between cues and targets across time. Red and blue colors respectively indicate relatively more versus less attentional resources allocated to probed visual field regions. The grayscale lines outline the contours of clusters found by a leave-one-out bootstrapped cluster permutation test, highlighting locations that consistently received more attentional resources than other locations across observers. (C) Pupil amplitudes per binned probe location (x axis) and timing (colors) averaged across observers. Shaded patches indicate standard errors from the mean. (D) Slope of linear fit to patterns in panel c per time bin. Negative and positive slopes indicate more attention to the cue and target, respectively. Shaded patches indicate standard errors from the mean. (E) The x-position of the center of mass of the clusters in B as a function of time. The thickness of the mean line extends to 95% confidence intervals of all lines from the leave-one-out bootstrap analysis (for details, see STAR Methods).

We presented single probes at different locations and time points across individual trials and measured the amplitudes of pupil responses time-locked to probe onsets (Figure 2A). To improve signal-noise ratios, amplitude time-series were averaged over trials per session, and within distinct space and time bins (number of maximum bins: horizontal = 16, vertical = 8, and time = 5; for details, see STAR Methods). This resulted in five maps (one per time bin) of the pupil amplitudes across space and time (left panel in Figure S1). It is important to note that the values in these initial maps were mostly dominated by the stronger perceptual (ocular) rather than more subtle covert attention modulations of the pupil amplitudes. More specifically, these maps appeared to only show a static effect of probe eccentricity with a foveal superiority. Due to cortical magnification and higher retinal receptor densities in the fovea, any probe shown near fixation evoked a stronger pupil response than probes far from fixation, independent of whether the probe fell within the focus of covert attention.^56^ As we were only interested in the modulating effects of covert attention, we removed such perceptual effects by baseline-subtracting a (static) map averaged across time bins per observer (center panel in Figure S1). This resulted in (dynamic) maps of covert attention per time bin averaged across observers, as shown in Figure 2B (for a video of an interpolated time-lapse, see Video S1), without the effect of probe eccentricity on pupil responses. Red-versus blue-coloured locations in each time-binned location of these maps indicate relatively more versus less attention, respectively. The pattern of changes in the “final” baseline-subtracted maps indicated that attention centers around the cue, then leaves the cue, to eventually shift and arrive at the target. This observation of shift characteristics was further supported by plotting pupil amplitudes averaged across the vertical axis of the maps, as shown in Figure 2C. The slope of a linear fit to each pupil amplitude pattern across space per time bin, reflecting left (negative slope) versus right (positive slope) biases of attention, also indicated an attentional shift from cue to target (Figure 2D; rmANOVA on slopes across time: *F*(4, 224) = 3.63, *p* = 0.007; slopes of first two time bins differ significantly from last two time bins (*p* < 0.05)).

Video S1. Interpolated time lapse of imaged covert attention maps of Experiment 1 This video shows interpolated data, demonstrating the movement of attention, as measured through enhanced pupillary responses to probes, from cue (start point) to target (endpoint) across time. Also see Figure 2B for binned instead of interpolated time lapses; the video was based on 12 instead of 5-time bins. Before interpolation, the spatial and temporal resolutions were up-sampled to 1600 by 800 pixels and 100 frames (25 Hz; 4 ms per frame). Values were interpolated across space and time with a cubic method. The C and 8 highlight the start (cue) and end target of the focus of covert attention. Time is shown in milliseconds.

The current paradigm also allowed us to roughly estimate temporal dynamics of attention, such as how long it took for attention to start moving after cue onset and to arrive at the target. The clusters detected with a leave-one-out bootstrapped cluster permutation test provided insights into attention’s movement profile. The overlaid contours in Figure 2B highlight significant attentional clusters (for details, see STAR Methods; for an alternative visualization, see Figure S2A), showing that attentional resources were widely distributed across space at multiple time points. However, as indicated by multiple contours lying closely together, the strongest attentional enhancements shifted their location. To quantify this, we calculated the center of mass of all significant clusters and plotted its horizontal position as a function of time (Figure 2E). While it is important to recognize that these clusters only provide rough estimates (rounded to tens and based on visual inspection), the profile suggests an arrival latency of 280 ms (based on the first time point after crossing the target’s center), which is in line with measurements by previous studies (see discussion). Previous studies could, however, not estimate the shift onset latency and speed of attention based on the period after shift onset. We here estimate these to be approximately 120 ms (based on the last time point before crossing the cue’s center) and 70 deg/s across 11.3 visual degrees, respectively.

It is important to note that covert shifts are typically accompanied by small overt gaze shifts away from fixation toward the target. We indeed observed small shifts of less than 0.3 deg (Figure S3). In theory, these could have slightly boosted pupil responses to probes presented slightly right from the cue location in the maps, but the permutation test highlighted no additional cluster at this location (see bottom panels in Figure 2B), and such small shifts can by no means explain the much larger shifts of >10 deg by covert attention.

### Experiment 2 – Covert shifts from fovea to periphery

**Figure 3:**
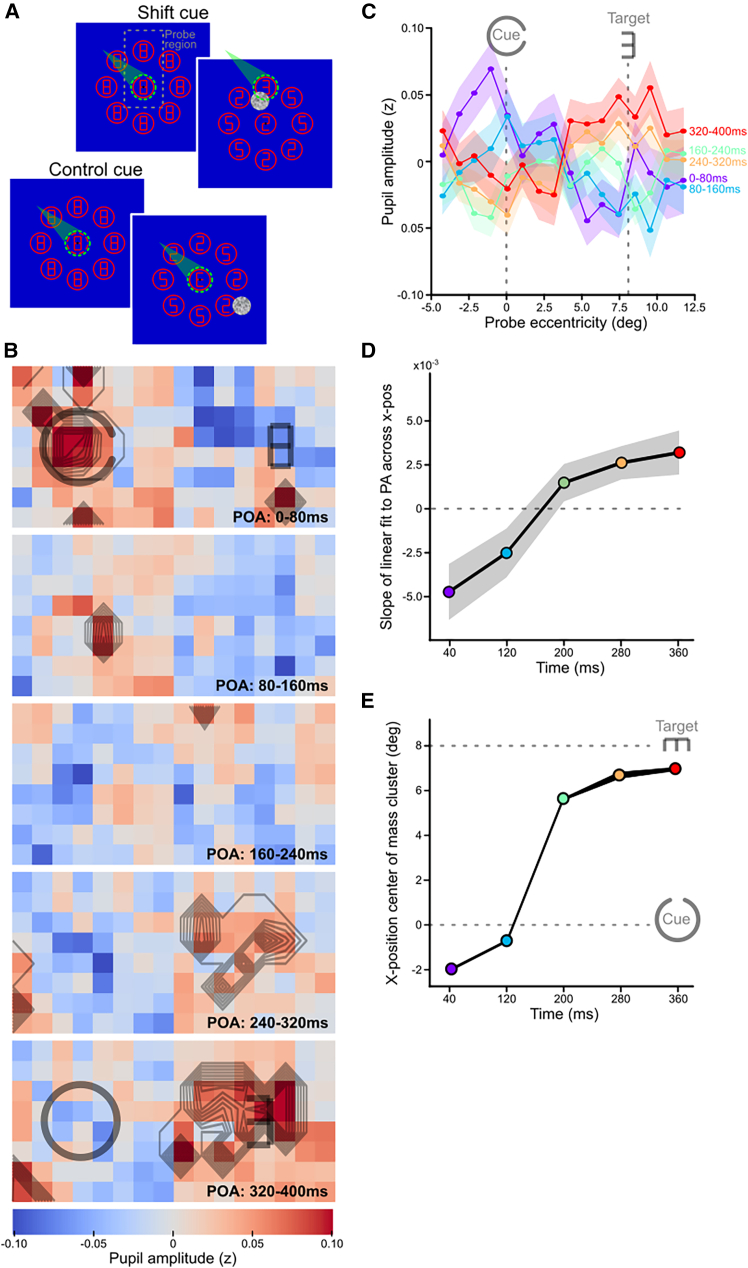
Attention imaging results of covert shifts of attention from foveal to peripheral locations (A) Procedure of experiment 2 in which all aspects remain identical to experiment 1, except that this time, observers were centrally cued at fixation. Observers covertly moved attention from foveal to peripheral locations, or maintained attention at the fovea in control trials. (B and C) Same results as in Figures 2B–2E, but now for Experiment 2.

The results of Experiment 1 suggest that covert attention shifts from cue to target locations, crossing intermediate sections at intermediate time points. This initial experiment tested how the attentional spotlight moves across multiple peripheral locations. However, in many other studies, covert attention must be re-deployed from the current overtly attended foveal location to the next potential peripheral target. To test how attention shifts in such scenarios, we conducted a second experiment with a slightly modified procedure and a new group of observers. Observers now had to shift attention from fixation to one of the 8 peripheral locations, and we added a control condition in which observers had to maintain attention at fixation (Figure 3A). The result of the attentional imaging analysis showed qualitatively similar spatiotemporal dynamics as in Experiment 1 (Figure 3B; for a video, see Video S2; for an alternative representation of clusters, see Figure S2B; for gaze control, see Figure S3B; for the baseline map, see Figure S4; for pupil responses per POA, see Figure S5A). The pattern of the distribution of attention between cue and target (Figure 3C), the bias in this distribution (Figure 3D; *F*(4, 228) = 5.94, *p* = 0.001; slopes of first two time bins differ significantly from the last three time bins (*p* < 0.05)), and the observed movement profile (Figure 3E) indicated comparable dynamics as in Experiment 1, with perhaps a slightly earlier arrival time, perhaps caused by the shorter distance (8 deg) between fixation and targets (note that the movement profile does not have sufficient resolution to draw strong conclusions).

Video S2. Interpolated time lapse of imaged covert attention maps of Experiment 2 Same as Video S2, but not for Experiment 2 (also see Figure 3B).

As expected, no spatial shifts of the clusters were observed in control trials, where the cue predicted the appearance of a central rather than a peripheral target. We did observe a general increase in cluster intensity and size over time, which was exclusive for this condition (Figures S5B and S5C). While we did not explore such magnitudal effects further in the current study, because our main interest was in the spatiotemporal component of attention, its observation highlights the potential of our method to measure increases (and decreases) of the intensity of attention in addition to spatial shifts.

### Experiment 3 – Measuring shifts at a higher temporal resolution

**Figure 4:**
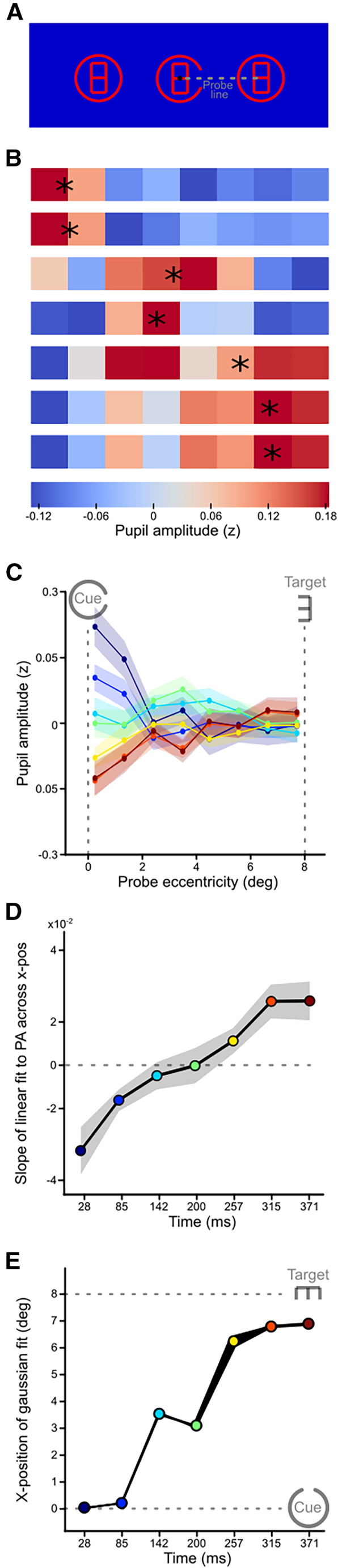
Results of covert shifts of attention from foveal to peripheral locations with probes confined to the horizontal meridian (A) Procedure of Experiment 3 in which all aspects remain identical to Experiment 2, except that this time, probes could only appear on the horizontal meridian (dotted gray line) either left or right from fixation. (B) Heatmap of pupil response amplitudes, averaged across observers, as a function of eight probe eccentricities (i.e., distance from fixation) across seven time periods of probe presentations (colors). Asterisks indicate the peak position of a Gaussian profile fitted to the data averaged across observers, for each time period. (C–E) Same as in Figures 3C–3E, but now for Experiment 3.

The previous two experiments enabled the imaging of attentional shifts at a relatively high spatial resolution and temporal resolution. The calculated shift properties were, however, based on a limited number of time bins. To improve the temporal resolution, we conducted a third experiment in which we sacrificed vertical spatial resolution for better temporal resolution. Probes only appeared on the horizontal meridian between fixation (and cue), and peripheral targets only appeared either left or right from fixation (Figure 4A). We first plotted pupil amplitudes as a function of probe eccentricity per time bin (Figure 4B). Similar to the previous experiments, changes in the slope of the distribution of attention across time showed that covert attention first focused on fixation before eventually ending at the target via an intermediate location (Figures 4C and 4D; *F*(6, 162) = 8.65, *p* < 0.001; slopes of first three time bins differ significantly from the last two time bins (*p* < 0.05); for gaze control, see Figure S3C). Next, to trace more closely how the focus of attention spreads across space and time, we determined the spatial location of the peak of a fitted Gaussian distribution (Figure 4E; also see asterisks in Figure 4B). This resulted in a movement pattern similar to those observed in the previous two experiments. As in Experiment 1–2, the observation of a relative increase of attention at intermediate locations and time points suggested that attention shifts across space.

## Discussion

This study implemented a pupillometric method to enable the imaging of covert shifts of attention at a relatively high spatial and temporal resolution. The approach allowed us to track how covert attention shifts between locations in three separate experiments. First, both the analysis of biases in the distribution of attention and the detection of attention clusters revealed that attention was enhanced at changing locations across time, starting with strong enhancements around the cue, followed by enhancements at intermediate locations between cues and targets, ending with enhancements around the target. We thus observed a shift of attention, despite the absence of lines or objects that connected cues and targets and could potentially bias and guide the position of attention. Second, the movement profile of the attentional enhancement roughly indicated when attention departed from the cue and arrived at the target.

The passing of attention through intermediate points is in line with previous work on curve tracing.^33^^,^^34^^,^^35^^,^^39^ However, the curve tracing literature has not fully resolved whether attention (1) spreads and extends over time, maintaining attentional resources at the start position, but gradually adding resources to intermediate and end sections, or (2) moves across space as a confined local spotlight that first leaves fixation to eventually end up at a target. The observed shifts in the current experiments align better with the latter account, mainly because we observe less attentional enhancements around the cue at later time points. In contrast to curve tracing studies, in our stimulus design, the attentional start- and endpoints are not interconnected, meaning that no traces or objects could have guided attention. This perhaps explains the difference between current and previous results. Our study further demonstrates that the pupil imaging technique enables the continuous measurement of attention across space, that is, at the start location, the end location, and in between. The technique is thus a viable tool to measure differential distribution of attentional resources across time and space at a high enough resolution to capture fast attention dynamics.

In line with the “mental spotlight” account of attention,^20^^,^^21^^,^^22^ we observe that shifts start with a local enhancement around the start location, and this enhancement moves toward the target across time, passing through intermediate locations while maintaining focus. These results confirm that the metaphor of the focus of attention as a spatially confined mental spotlight is correct and additionally reveal the moving rather than spreading nature of its spatial displacement, at least in the absence of objects that may guide attention.

Besides these spatiotemporal dynamics, our paradigm also has the potential to provide insights into the temporal aspects of attentional shifts. In contrast to previous behavioral work measuring only arrival times, we could also roughly estimate the movement onset latencies and the speed of shifts of the attentional spotlight after observing static and dynamic periods in all three experiments. Previous research predicted shift durations of around 100-300 ms,^23^^,^^24^^,^^25^^,^^26^^,^^27^^,^^28^^,^^29^^,^^30^^,^^31^^,^^32^ although the exact timing varies substantially depending on the setting of a variety of factors, such as whether shifts are made voluntarily or involuntarily^31^ and the type of attention paradigm employed.^57^ The here observed arrival times (200-300 ms) fall within this range, although being biased toward the upper bound. This delay may be caused by the probe flashes, perhaps distracting attention and slowing its movement onset or speed.^23^ Our results further appear not to be fully in line with previous work showing that peripheral cueing results in faster arrival times.^23^^,^^58^ The observed visual shifts also tend to be slower than auditory shifts.^59^ However, we have kept cue saliency equal across cueing conditions, while these previous studies have used much more salient cues for the peripheral than central conditions. It is not unlikely that more salient exogenous cues evoke faster shifts, but future studies will have to investigate how factors such as shift distance and cue saliency affect shift latencies and speeds. Future studies may also investigate more closely whether attention moves gradually or makes multiple ballistic jumps.

Future work employing our pupillometric paradigm can now focus specifically on multiple timing aspects of attention through a more fine-grained temporal analysis of shift onsets, speeds, and offsets, and attentional dwell times. For example, attention can be voluntarily directed to points in time,^60^ and our paradigm permits us to investigate how such attentional enhancements evolve over time. Another aspect that asks for a more thorough investigation concerns the distinction between serial and parallel attentional probing.^61^ It is well possible that the attentional focus is split into multiple spotlights that independently move to distinct locations in parallel. Furthermore, comparing pre-saccadic shifts of attention with covert shifts of attention independent of eye movements may also foster interesting results in future studies. For example, pre-saccadic attention arrives much faster (60 ms) at target locations than voluntary covert shifts of attention,^62^ and we wonder which movement properties (e.g., movement onset latency or movement speed) underlie this sped up. Pre-saccadic attention is likely a different form of attention and controlled by different brain processes,^63^ as confirmed by electrophysiological studies that show that typical components of brain activity, normally evoked during covert shifts, remain mostly absent during pre-saccadic shifts.^64^^,^^65^ Conversely, future efforts could also try to enhance the spatial resolution—at the expense of temporal resolution—to visualize the exact shape and size of the spotlight under varying conditions,^47^^,^^66^^,^^67^ for example, to confirm the supposed existence of an inhibitory ring encircling the spotlight, i.e., a Mexican hat profile.^27^^,^^46^^,^^68^^,^^69^^,^^70^^,^^71^^,^^72^^,^^73^^,^^74^; Such spatial tuning even generalizes to the feature domain,^75^ as feature-based attention mostly shares the neural system of spatial attention.^76^ A shift of focus between features, such as diverging colors, may also follow a zoom-in and zoom-out mechanism with a spotlight that is dynamic in size.^77^ Another interesting aspect to study would be to investigate how attention moves toward or spreads over multiple targets,^78^ for example, by comparing pupil responses to probes presented between two simultaneous targets. Lastly, our findings may shed light on how individuals vary in spatiotemporal properties of the spotlight and how these relate to attentional processing styles and disorders such as autism spectrum disorder (ASD), attention-deficit (hyperactivity) disorder (AD(H)D), and neglect. It is important to note, however, that the scrutiny of individual differences in attentional movement profiles will require substantially more trials than in the current study.

To conclude, we discovered new properties of attentional shifts by applying an effective pupillometric imaging method. We showed that the focus of attention shifts across locations with a specific speed profile. This study opens up new avenues in investigating yet-to-be discovered effects on spatiotemporal properties of attention in a detailed manner.

### Limitations of the study

It is important to note that the here-reported temporal estimates of attention were rough approximations, based on visual inspection rather than statistics, and limited by the chosen temporal resolution. These limitations prevent us from making strong statements about the speed of attentional shifts. Fortunately, this does not limit future studies from increasing the temporal resolution, likely at the cost of spatial resolution. Our paradigm is flexible in the sense that the time window of appearing probes can be narrowed down and focused on, for example, the 75-125 ms window to more accurately detect attentional departure times. Vice versa, the spatial profile of the focus of attention may also be improved by using smaller probe sizes, though with the cost that these produce less robust pupil responses. Future studies can thus focus on specific properties of attention to determine the exact onset times, arrival times, and shape of the attentional spotlight.

One other point of critique of our approach relates to the way we measured attention. While previous research mostly measured attentional effects using behavioral or neural measures, we measured constrictions in pupil size. One may question whether modulations of the pupil response tap into the same attentional system as traditional reaction times and detection/identification accuracy measures in cueing, search, change detection, and other attention paradigms. However, several findings support the idea that pupillometry measures similar aspects of attention as behavioral studies. Besides the overlap between current and previous shift-timing findings and predictions that pupil constrictions should become stronger when a visual event, like the presentation of a probe, happens to fall within the focus of attention,^48^^,^^79^ there is also substantial converging evidence from cognitive and neuroscientific studies.^48^^,^^52^^,^^80^ First, subtle allocations of endogenous and exogenous covert spatial attention to light increments and decrements concurrently change pupil responses.^49^^,^^50^^,^^51^^,^^53^^,^^54^^,^^55^ Second, such attention-modulated pupil responses correlate with behavioral performance.^51^ Last, pupil responses causally depend on activity in two brain areas involved in attentional orienting, namely the superior colliculus^81^^,^^82^^,^^83^ and FEF.^84^^,^^85^ Despite being a peripheral physiological measure, pupil responses to sudden probe onsets thus directly reflect attentional operations that emanate from neural connections between cortical and deeper subcortical nuclei, such as the superior colliculus.^48^^,^^86^

Not only does endogenously controlled covert attention modulate pupil responses. In fact, the amplitude of responses also depends on stable properties of the visual system, such as the distribution of receptors in the eye and the cortical magnification factor that enhances foveal (i.e., central) vision. Such ocular and perceptual factors directly link to effects of stimulus eccentricity^56^^,^^87^ and salience,^88^ suggesting that any stimulus that is processed more profoundly evokes stronger pupil responses. Therefore, one could argue that the early and late enhancements of responses to probes presented around the cue and target, respectively, are driven by attentional capture or salience triggered by the local changes within the cue and target itself, rather than the probes presented within the focus of endogenous attention. It should be noted, however, that the cues and targets evoke *distinct* and *weaker* pupil responses before and after the much stronger pupil responses to the probes (see small constrictions around 0.75 s and 1.25 s in Figure 2A). The here visualized attentional shifts are specifically based on pupil responses to probes, while responses to other visual events were deliberately reduced using equiluminant stimuli. Two other reasons why the current results cannot be explained by such capture effects are that we observed (1) a *relative* shift of a *local* enhancement over time and (2) a local enhancement at intermediate time points (i.e., around 150-200 ms). At these intermediate time points, probes are presented across the entire spatial span between cues and targets. Each of these probes exogenously captures attention, therefore, is unable to explain why only probes presented at intermediate locations and intermediate time points evoke enhanced pupil responses.

One may argue for two other alternative explanations for the observed local enhancements at intermediate time points and locations. First, participants may have learned that probes appear on average between the cue and target (i.e., the center of mass of the distribution of probe locations) and that this consequently attracts more attention to intermediate locations. Although we deem it unlikely that participants have had enough residual attention to track such irrelevant probe statistics while also following cue directions and identifying targets, such an effect should have resulted in enhanced attention at intermediate locations at any time point. However, we observe the strongest enhancements of attention at intermediate locations only at intermediate time points. Second, when attention shifts abruptly at varying time points rather than gradually per trial, the mere averaging of attention across trials may theoretically lead to an apparent increase of attention at intermediate locations and time points. However, such a pattern is only expected if the focus of attention is widely distributed across space, with relatively higher weights at intermediate attended locations than unattended locations. In contrast, we find clear local enhancements rather than a spread of attention, as confirmed by the permutation tests, rendering it unlikely that the here observed gradual shift is an averaging confound.

Perceptual and ocular effects also enhance the pupil responses, resulting in an additional gradient of enhanced responses to probes presented near fixation. However, these effects are stable and not dynamic across time, and we filtered out such eccentricity effects by subtracting a static baseline map averaged across time. In sum, we dissociate (1) a bottom-up, static, and perceptual (ocular) or exogenous attention component from (2) an additional dynamic and modulatory component that depends on whether a probe falls within the endogenously controlled focus of attention. It should be noted, however, that future studies could potentially improve attention measurements by disentangling multiple (perceptual and attentional) pupillary components through the modeling of pupil responses to distinct impulse events.^79^^,^^89^

We performed no analyses on potential effects of sex, gender, and other demographics, because (1) no scientific literature exists that suggests that pupil responses to probes should vary across demographics, (2) we tested a relatively homogeneous sample (mostly students), and (3) power of the current sample would drop in reliability if we were to perform between-group (e.g., male vs. female) comparisons. The absence of these analyses limits the study’s generalizability to other (e.g., elderly) populations.

## Resource availability

### Lead contact

Further information and requests for resources and reagents should be directed to and will be fulfilled by the Lead Contact, Marnix Naber (marnixnaber@gmail.com).

### Materials availability

This study did not generate new unique reagents or other materials.

### Data and code availability

• Data: Data that support the findings of this study are publicly available as of the date of publication. A link to the repository can be found in the key resources table.
• Code: All original code has been deposited at the Open Science Framework (OSF) and can be used to run the experiments and to analyze the data of this study. A link to the repository can be found in the key resources table.
• Additional information: Any additional information required to reanalyze the data reported in this paper is available from the lead contact upon request.

## Acknowledgments

We thank Christoph Strauch and Sebastiaan Mathot for discussions about pupil constriction responses and how they relate to attentional orienting. We thank Marit Reurink, Isa Robben, Sidney Van der Wel, and Loeka Van Diemen for help during data collection. This research was funded by a VICI Grant (#VI.C.211.011) from the Netherlands Organization for Scientific Research (NWO) to Stefan Van der Stigchel.

## Author contributions

All authors designed the experiments. Authors M.S. and LvdB programmed the experiments and collected the data. Authors M.S., M.N., and S.C. analyzed the data. Author M.N. wrote the draft, and all co-authors read, edited, and approved the final version of the manuscript for submission.

## Declaration of interests

The authors declare no competing interests.

## STAR★Methods

### Key resources table

**Table:** 

REAGENT or RESOURCE	SOURCE	IDENTIFIER
**Deposited data**		

Raw data, experiment psychopy code, and analysis code	OSF Storage	https://osf.io/tynpb/overview?view_only=63403226baa04367918783eea1924b4e.

**Software and algorithms**		

Python version 3.8.10	Python Software Foundation	https://www.python.org
Psychopy version 2022.2.4	Peirce and colleagues	https://www.psychopy.org/

**Other**		

EyeLink Eye Trackers (Eyelink 1000 Plus Tower mount)	SR Research	https://sr-research.com
OLED Television Screen	LG	https://www.lg.com/

### Experimental model and study participant details

#### Participants

Thirty-four (Age: M = 22.9, SD = 3.9; 25 females; 4 left-handed; All Dutch, Caucasian), thirty-three (Age: M = 22.6, SD = 3.0; 23 females; 3 left-handed; All Dutch, Caucasian), and twenty-seven (Age: M = 22.5, SD = 1.9; 16 females; 11 left-handed) observers participated in Experiment 1, 2, and 3, respectively. These sample sizes match or exceeds those of previous pupillometry studies on visual attention (e.g., Naber et al., 2013). Observers had either normal vision or used contact lenses to correct myopia or hyperopia. Observers wore no makeup such as mascara to prevent threshold interference for the detection of the pupil by the eye tracker. We recruited observers through an online recruitment platform (SONA systems LLC, Bethesda, MD, USA) of Utrecht University. During the recruitment process, potential observers were excluded if they reported to be diagnosed with an attentional disorder or photosensitive epilepsy. We compensated observers with either course credits or monetary means. The faculty Ethics board of Utrecht University approved this study before data collection (#24-0536) and observers signed informed consent forms for participation and the use of data collected.

### Method details

#### Apparatus

The experiments took place in a darkened room with black walls and a two-computer setup. The first computer used a dual-screen setup with one 55inch OLED television screen (LG) for displaying the stimuli (resolution: 1920x1080 pixels; frame rate: 100Hz; width: 145cm; viewing distance: 76.5cm) and another TFT screen (identical resolution and frame rate as the television) for monitoring experimental progression. PsychoPy Version 2022.2.4^90^; was used to build the experiment, and Python (Version 3.8.10) to run it. An observer’s head was positioned in a chin and forehead rest of an EyeLink 1000 Plus Tower Mount (SR Research, Mississauga, Ontario, Canada). The eye-tracker was connected to the second computer which utilized the SR Research software to run the eye-tracker code and display information to the experimenter about both gaze position and pupil size. Eye-tracker recording sessions preceded a 5-point (at screen centre and at each cardinal axis at 10 degrees eccentricity) calibration and validation procedure. A noise-reduced keyboard with haptically distinct response buttons ensured that observers could find the keys in complete darkness.

##### Stimuli and procedure – Experiment 1

The main experiment consisted of one or two sessions of 1250 trials, each consisting of 5 blocks of 250 trials. A trial consisted of four steps (see Figure 1B): the presentation of (1) a cue pointing towards the target location to which observers covertly shifted their attention, (2) a probe to evoke a pupil response, (3) a target to be attended and three non-targets to be ignored, and (4) an intermittent screen during which observers reported the target’s identity. As the probe-evoked pupil response (2) was the main measure of interest, we aimed to minimize effects of luminance and contrast of the other task-relevant stimuli (i.e., cue and target) on the pupil responses. To accomplish this, we made cue and target stimuli (dark red) equiluminant to their background (dark blue; physical luminance: 14.8 candela per m^2^) using a flicker fusion procedure preceding the main experiment. The procedure consisted of the presentation of a flickering red circle (20 hz; 5 degrees in diameter) on top of a dark blue background. An observer adjusted the colour intensity of the red circle by moving a slider with a computer mouse until they observed minimal flicker (a subjectively equiluminant stimulus appears to flicker less strongly).

After this procedure, participants viewed a slowed down visualization of a single trial with instructions, practiced a couple trials, and then started the main experiment. During each trial, observers continuously fixated a dot (black; 0.5 degrees in diameter) at the centre of the screen. Four red outlines of circles (5 degrees in diameter; 0.4 degrees line thickness) presented at 8 degrees eccentricity at the cardinal axes served both as cues and placeholders for digital “8s” that could change into a target or non-target. A portion of one of the four circles’ outline (1 visual degrees in length) was removed for 500 ms, creating a C-shaped arc with the opening pointing towards a target’s placeholder at a clockwise or counter-clockwise direction (randomized across trials). The onset of this opening cued observers to start moving their attention covertly from the cued location to the target location. While performing the shift, a high-contrast probe (3 degrees in diameter; white noise spatial frequency content: 2-4 cycles per degree) appeared at a location (randomly varied across trials) for 100ms within a rectangular space (20 by 10 degrees in size) spanning around the cue and target. A probe’s onset asynchrony (POA) with respect to a cue onset was randomly chosen from a range between 0 and 400ms per trial. This range was based on previous attention timing studies.^23^^,^^24^^,^^25^^,^^26^^,^^27^^,^^28^^,^^29^^,^^30^ The cued “8” temporarily changed into a target (a “3” or an “E”) thereafter (400-600 ms with respect to cue onset) in parallel with the uncued “8s” that changed into non-targets (“2s” and “5s”) for a duration of 500ms. Observers had to respond within 1500ms after target onset by pressing a keyboard button corresponding to the target’s identity.

Observers partook in either one session (n = 11; did not show up for session 2), or two sessions (n = 23) conducted on separate days, resulting in 57 sessions in total. A practice block of 80 trials preceded the main experiment to (1) get observers acquainted to the task and (2) run a staircase procedure to ensure a similar level of performance across observers (aimed for a target identification accuracy: 87.5%). The staircase decreased or increased the thickness of the digits by 0.05 degrees after each correct or incorrect answer, respectively. The resulting level of task difficulty forced observers to use attention to boost their peripheral vision to identify targets.

Note that the experiment did not include invalid cue conditions (i.e., the observer is cued to covertly shift attention to a non-target rather than target location). Such conditions are normally included to demonstrate that covert attention is successfully deployed and boosts target identification performance in valid trials as compared to invalid trials. For the sake of the duration of the experiment we excluded such trials and trusted on the proven effectiveness of the current design in manipulating covert attention as demonstrated in numerous previous studies with similar designse.g.^9^^,^^91^^,^^92^^,^^93^

##### Stimuli and procedure – Experiment 2

All aspects of Experiment 2 were identical to Experiment 1, except for the direction of attentional shifts, the addition of control trials, and the implementation of a staircase that continuously adjusted target presentation duration throughout the experiment. Instead of moving covert attention between peripheral targets, covert attention moved from foveal fixation to one of eight peripheral targets. This allowed us to assess whether the properties of peripheral-to-peripheral shifts found in Experiment 1 generalized to foveal-to-peripheral shifts. Also, twenty percent of the trials consisted of no-shift trials in which the cue (a circle outline with 8 openings pointing to all placeholders) instructed observers to maintain attention at fixation. These trials constituted a control condition to inspect whether attention did not move across space when no shift was required. Last, a staircase procedure lengthened the duration of targets with steps of 24ms after incorrect trials or shortened the duration with 3ms after correct trials (start duration: 320ms; mean target duration across trials, averaged across observers: M = 326 ms, SD = 188 ms, range = 42-615 ms) during the main experiment (i.e., not only during the practice block) to keep task difficulty equal across the experiment. To maintain equal trial durations across observers, observers had a total of 1300 ms to respond to targets after onset. Observers partook in either one session (n = 9) or two sessions (n = 24) conducted on separate days, resulting in 57 sessions in total.

##### Stimuli and procedure – Experiment 3

All aspects of Experiment 3 were identical to Experiment 2, except for the number of potential target locations and locations of probes. Targets and probes were only shown left or right from fixation. Probes could only appear between fixation and target centre on the horizontal meridian. By sacrificing the 2-dimensional aspect of the spatial imaging, as done in Experiment 1 and 2, Experiment 3 increased the spatiotemporal resolution, though only in 1-dimensional space (i.e., only the horizontal, not the vertical space). All observers partook in one session of 1250 trials.

### Quantification and statistical analysis

The amplitude of the pupil response to the onset of probes served as the main dependent measure of attentional resources. We performed an event-related pupil response analysis in which each probe onset served as an event. The same analysis was performed with cue onsets as events as well (see Figures 1C and 2A), but only to show that the strongest pupil responses were time-locked to probe onset.

First, before segmenting pupil responses, missing pupil diameter values measured during blink periods were interpolated using a “pchip” algorithm. Blink onsets were detected by looking for abrupt pupil constrictions with a speed value (2ms window) of 4 standard deviations above average. Blink offsets were detected by searching for a dilation following a blink onset with a speed of 2 standard deviations above average. The resulting blink segments were diluted by 50 ms to take into account potentially late-onset threshold crossings of speeds of constrictions.

Next, 2000 ms segments of pupil size and gaze position traces, starting at probe onset, were concatenated across trials. We calculated pupil response amplitudes by subtracting the minimum pupil size within a window of 250 ms to 1750 ms (for the timing of maximum constriction, see Figure 2A) from baseline pupil size (average in a 0-250 ms window) per trial.

A trial was considered faulty if the trial data showed (1) gaze locations to far away from fixation, (2) relatively few (not yet interpolated) pupil size samples (e.g., caused by too many blinks), (3) deviating pupil response amplitudes (e.g., very large responses caused by weak blinks that escaped blink detection), and (4) incorrect target reports indicating that attention was not allocated correctly. For the former 3 aspects, we used a relative selection rule rather than an arbitrary threshold, consisting of a deviation of four standard deviations from the mean. Manual inspection of the resulting thresholds and data of the removed trials confirmed that these settings effectively removed of unwanted trials. Trials with broken fixations (Exp. 1: M = 1.1%, SD = 0.5%; Exp. 2: M = 1.1%, SD = 0.6%; Exp. 3: M = 0.9%, SD = 0.4%), not enough samples (Exp. 1: M = 0.5%, SD = 0.6%; Exp. 2: M = 0.3%, SD = 0.4%; Exp. 3: M = 0.4%, SD = 0.6%), exceptionally large response amplitudes (due to artifacts; Exp. 1: M = 0.9%, SD = 0.8%; Exp. 2: M = 1.2%, SD = 0.9%; Exp. 3: M = 0.4%, SD = 0.3%), and incorrect target identifications (Accuracy in Exp. 1: M = 89%, SD = 9%; Exp. 2: M = 87%, SD = 4%; Exp. 3: M = 85%, SD = 5%; see Figure S6) were not included in the analysis. A post-hoc fixation analysis was performed to ensure that the observed attentional shifts of 8 deg could not be explained by the much smaller shifts in gaze (<0.3 deg during the shift of attention in 0-400 ms; see Figure S3).

Counterclockwise and clockwise shifts of attention were pooled independent of the location of the target and cue by converting the coordinates of the probe locations to a single two-dimensional reference attention map (for an example, see dotted lines in Figure 1C). Next, to take into account large variability in pupil responsiveness across individuals and ease comparison across individuals, we z-normalized pupil response amplitudes by subtracting the average amplitude and dividing by the standard deviation across trials per observer. Furthermore, the pupil typically responds stronger to stimuli presented in the upper visual field i.e., a visual field anisotropy^51^^,^^56^; which adds unwanted variance to the data. Before visualizing the shifts of attention based on pupil response amplitudes, we removed visual field anisotropies by correcting the amplitude values through subtraction of the difference in the pupil response amplitude, averaged across trials per observer, between top and bottom visual field target locations. This resulted in equal amplitudes for probes presented in the top and bottom half of the visual field.

To create two-dimensional maps of amplitudes, we averaged the pupil response amplitudes corresponding to each time point per space segment, capturing the spatial distribution of responses over the screen for each specific moment. Rather than binning the segments using a fixed-width (or equal-width) procedure with somewhat arbitrary spatial and temporal bin boundaries, we applied a nested (hierarchical), and less arbitrary, binning procedure (see Figure S7). Multiple maps were produced using a range of segment (i.e., bin) sizes, each creating a map with different spatial and temporal resolutions. We set the maximum number of segments to 3-8 (steps of 1; 0 in Exp. 3) for vertical, 6-16 (steps of 2) for horizontal, and 3-5 (steps of 1; 3-8 in Exp. 3) for time, which resulted in averages based on a minimum of 3 to 4 amplitudes (i.e., trials) per segment at the highest resolution (setting the number of segments higher would have resulted in very low signal-to-noise ratios) and a minimum of 40-50 amplitudes at the lowest resolution. The number of amplitudes per bin varied slightly across bins as probe locations and timings were randomly drawn from a uniform distribution. The map consisted of the average across all the maps with different segment sizes, with a final segment (i.e., voxel in the images) width of 1.33 and 1.13 degrees in visual angle for Experiment 1 and 2, respectively. These maps did not yet reflect only effects of attention, but also included a perceptual (including ocular) effect of eccentricity on pupil response amplitudes i.e., the pupil responds stronger to items appearing in the fovea than periphery^56^; This effect was removed by subtracting the map averaged across time bins (see Figure S1), resulting in a final attention map that conserved relative, dynamic, and endogenous effects of covert attention.

To investigate biases in the distribution of attention towards the cue or target, we averaged pupil amplitudes per bin of the probes x-positions (i.e., ignoring the y-position in Experiment 1-2) and plotted these per time bin (Figures 2C, 3C, and 4C). Next, we fitted a linear regression line to these patterns per time bin with the slope of each line indicating attentional biases per time bin (Figures 2D, 3D, and 4D). A repeated measures ANOVA and post-hoc t-tests were performed on the slopes to see whether and across which time points slopes differed.

For Exp. 1 and 2 we performed a 2-dimensional nonparametric cluster-based permutation test^94^ to investigate how focal attention shifted across space in Experiment 1 and 2. Notably, because such tests compare z-scored values against 0 (thus for data centering around 0), this statistical procedure is likely less sensitive to only positive spatial variations in the maps (i.e., the focus of attention). To correct this limitation we baseline-corrected the spatial maps by subtracting the 45^th^ percentile of all values within each map per session. This was done parametrically because subtracting too large values would make it impossible to detect any clusters whereas subtracting to small (or negative) values would lead to an inclusion of all pixels in a cluster. Importantly this procedure cannot lead to the artificial generation of clusters at specific locations because single values were subtracted from all pixels within a map, thus preserving the relative values between pixels. Next, we performed two sided t-tests per location in the map to identify pixels representing z-scored pupil amplitudes that significantly and positively deviated from 0. We then summed up the t-values of all pixels within an individual cluster (i.e., all significant pixels connected by an edge or corner) to determine its t-mass. To test if the observed clusters t-mass was to be expected under the null hypothesis (H0: the original data is symmetrically distributed around 0) we generated a null-distribution of t-masses. This null distribution was generated by changing the sign of all pixels for a random number of maps. This procedure was repeated 1000 times, each time detecting clusters of neighbouring significant pixels and summing their t-masses. Last we compared the veridical cluster-level t-masses to those of the null-distribution and tested if their mass exceeded the 97.5% quantile (i.e., an alpha level of 0.05). For Experiment 3, a Gaussian profile was fitted to the pattern of pupil amplitudes, averaged across observers, to detect the probe location that evoked the strongest (peak) amplitude. The confidence intervals, as shown in Figures 2E, 3E, and 4E, were based on outcomes from the above explained analyses using a leave-one-out bootstrap procedure.
