# Supplementary material for: Imaging the spatiotemporal dynamics of covert attention with a pupillometric probing paradigm
Source: iScience. 2026 Mar 18;29(4):115386. doi: 10.1016/j.isci.2026.115386 (PMC13062523; doi:10.1016/j.isci.2026.115386)
Supplement: Document S1. Figures S1–S7 [file mmc1.pdf]

iScience, Volume *29*

Supplemental information

Imaging the spatiotemporal dynamics of covert attention with a pupillometric probing paradigm

Marnix Naber, Marinos Savva, Lotte van den Berg, Stefan Van der Stigchel, and Samson Chota

Supplementary materials

Imaging with pupil responses reveals the spatiotemporal dynamics of covert attention.

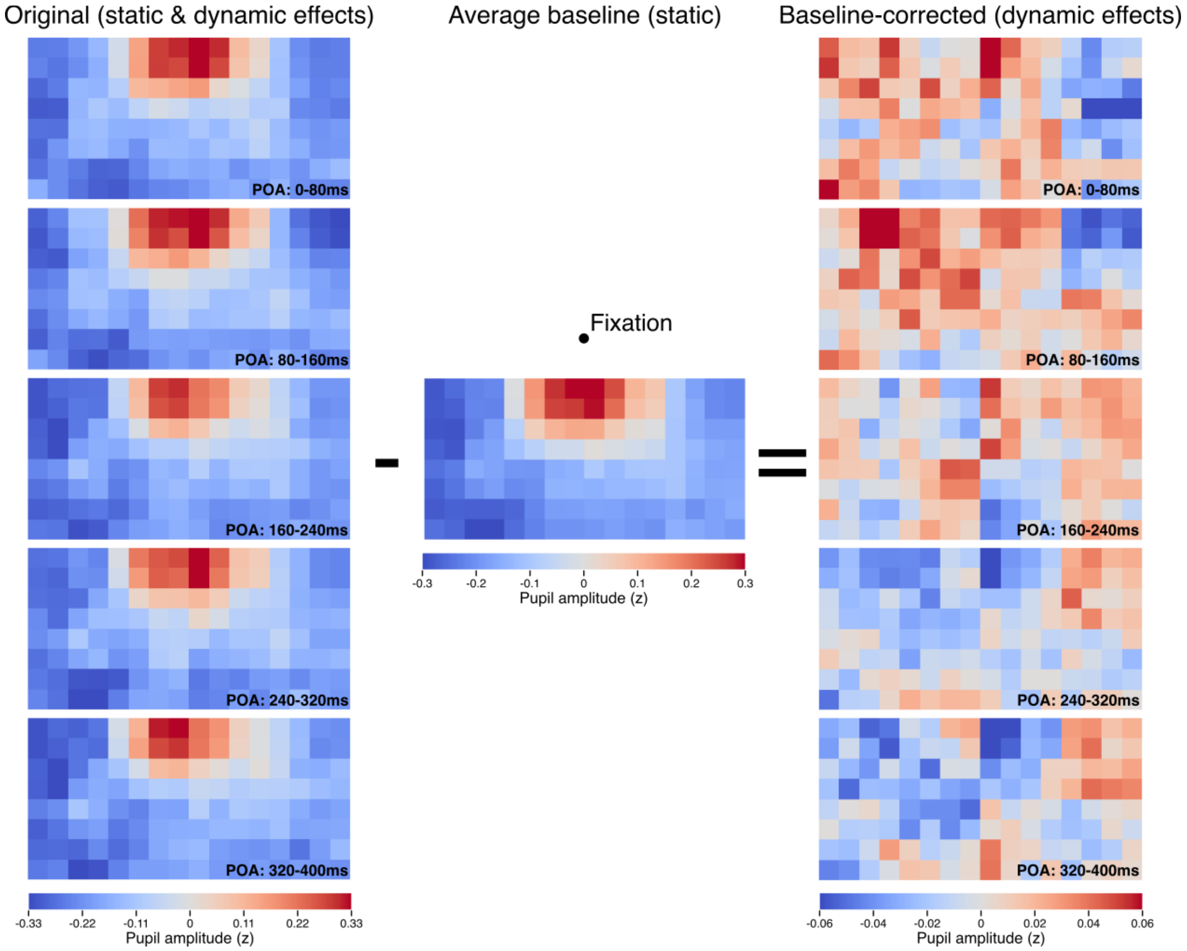

Supplementary figure S1. Imaged perceptual maps of Experiment 1. *The images in the left panel show strong perceptual modulations on pupil response amplitudes per time bin. Pupil response amplitudes decrease as a function of probe eccentricity (i.e., distance from fixation; black dot). The centre map, showing the session-average of the panels on the left, serves as a time-invariant (static) baseline. The maps on the left are subtracted by the baseline map in the centre to end up with the maps on the right displaying the dynamic effects of covert attention.*

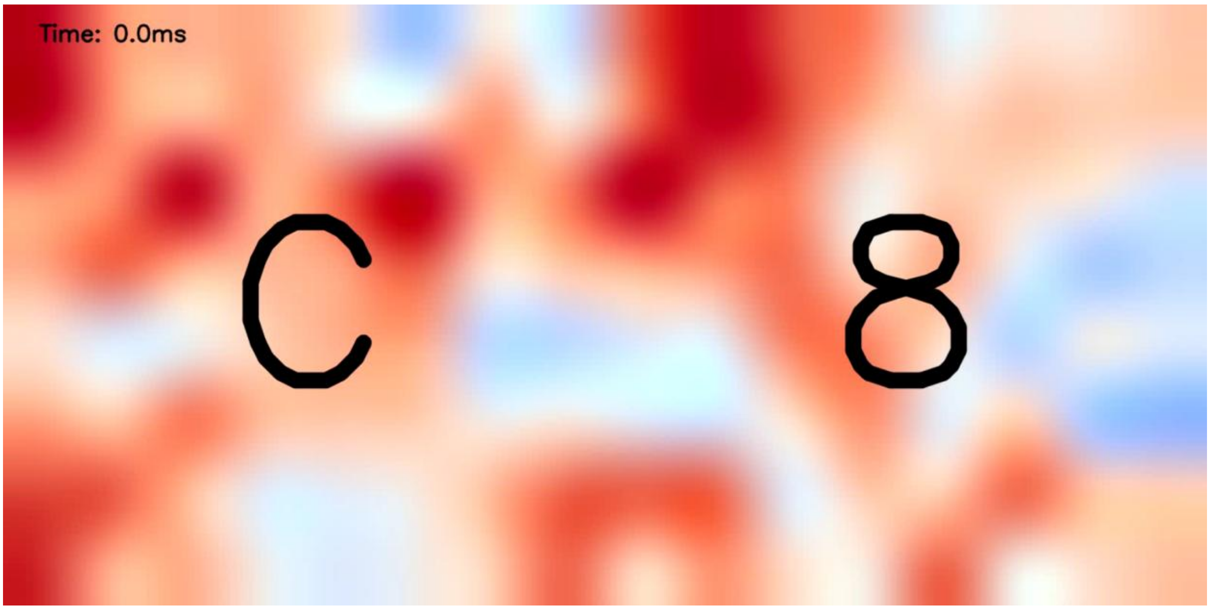

Supplementary video 1.

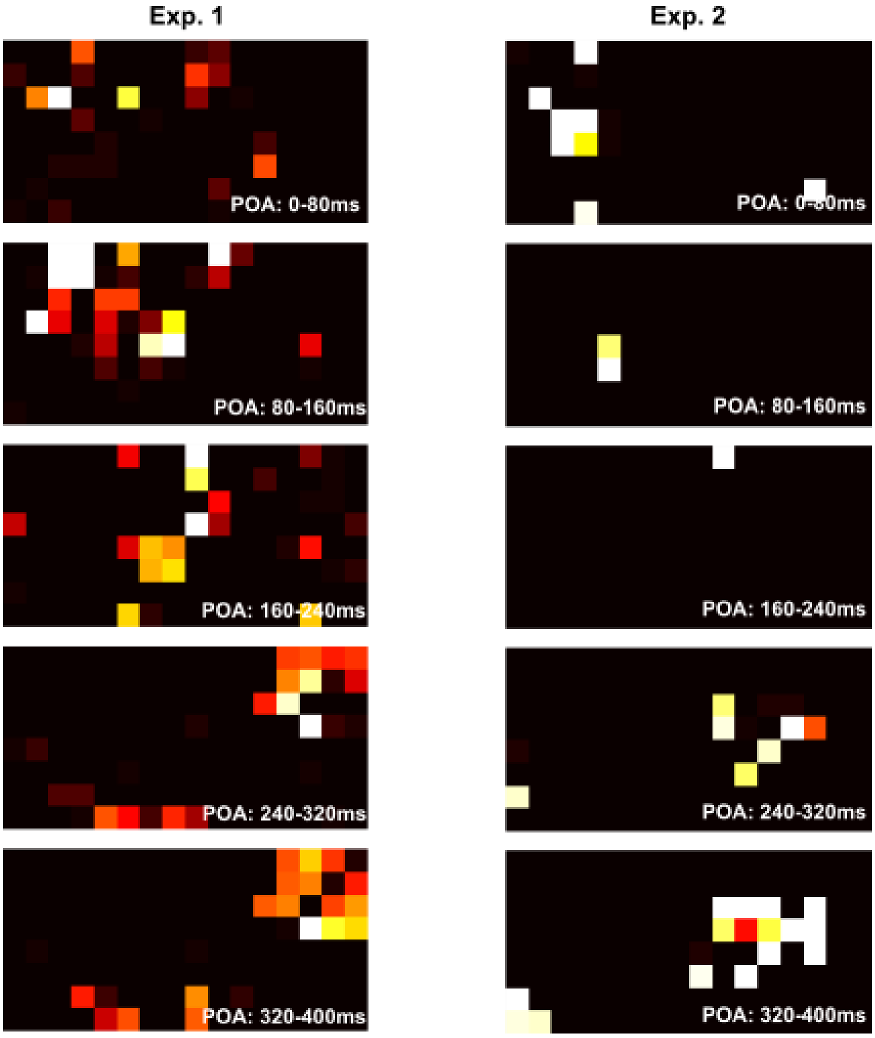

Supplementary figure S2. Cluster detection from leave-one-out analysis. *The heatmap represents the sum of all positive clusters detected by the permutation test per time bin (0 = no clusters detected, white = clusters detected in all leave-one-out iterations). The contours shown in Figure 2 and 3 in the main paper were based on these plots.*

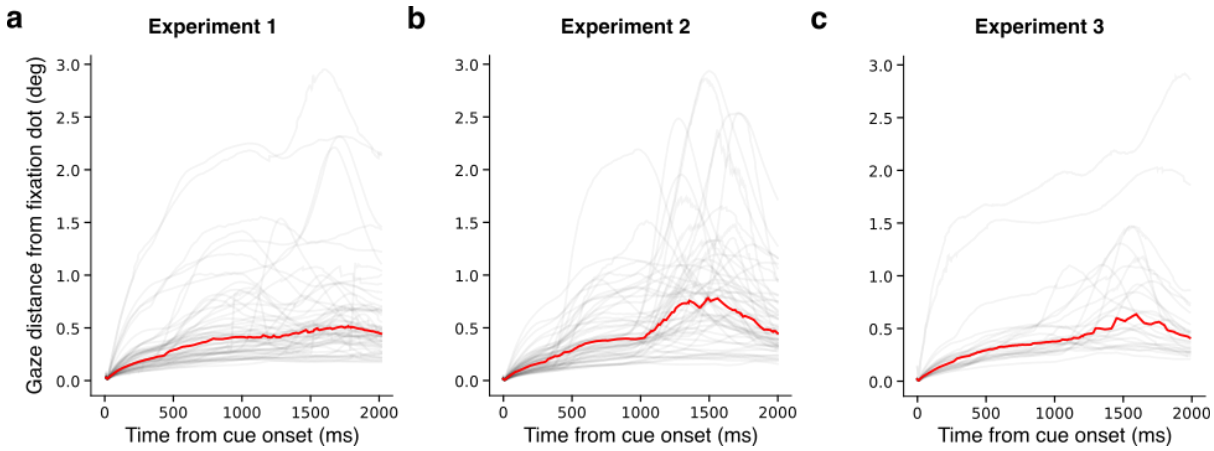

Supplementary figure S3. Gaze fixation analysis per experiment. *Gaze distance from the fixation dot at the centre of the screen as a function of time from cue onset. Grey lines depict the average across trials per observer and the red line shows the average across observers. Attentional shifts completed around 300ms, at which the gaze deviated less than 0.5 deg from fixation. Note that deviations could also be in directions away from targets that were located at 10 deg from the fixation dot.*

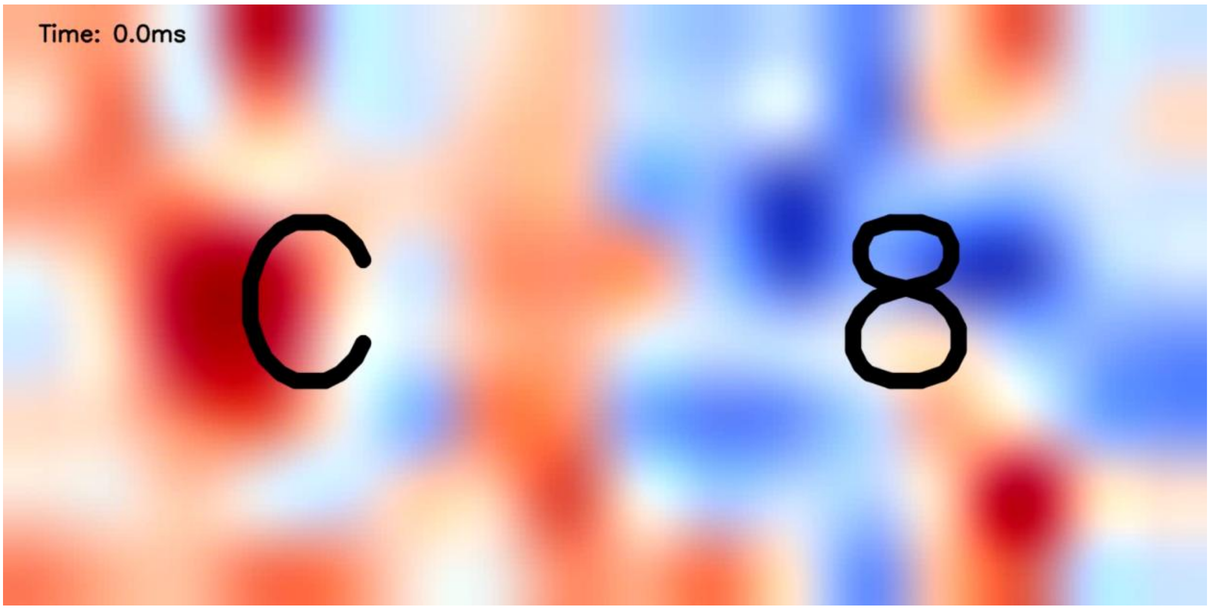

Supplementary video 2.

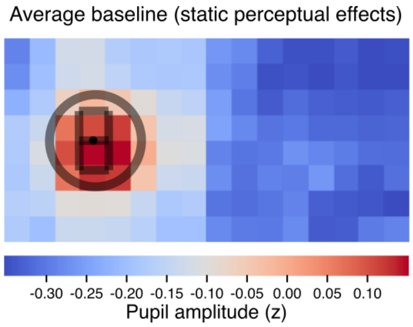

Supplementary figure S4. Imaged perceptual map of Experiment 2 averaged across time. *Same as figure S1 but now for experiment 2.*

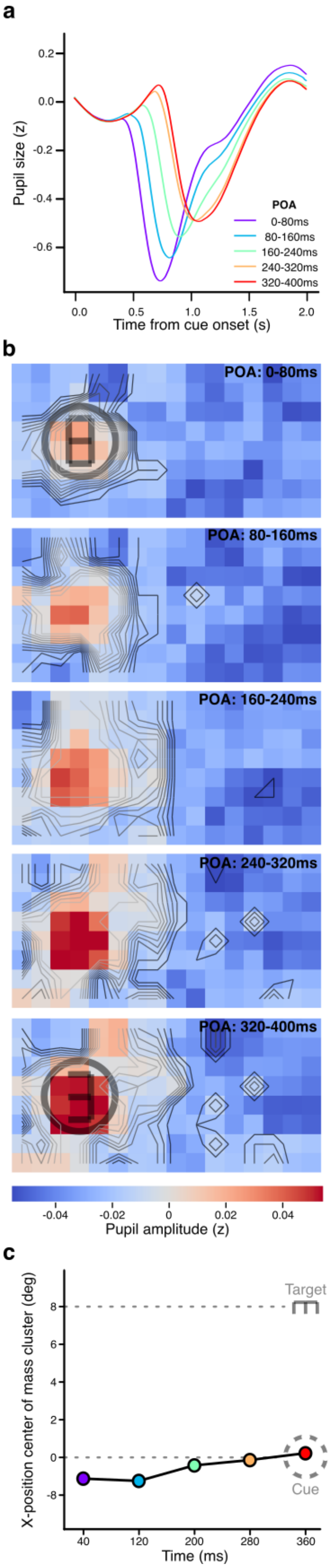

***Supplementary figure S5. Pupil responses and imaged attention maps of Experiment 2 control trials results. a**, Pupil responses per probe onset asynchrony bin, as in **figure 2a**, but now for Experiment 2. **b**, Attention maps for control (no-shift) trials with clusters during which the focus of attention had to be maintained at fixation. **c**, The x-position based on the centre of mass of the clusters in panel b as a function of time.*

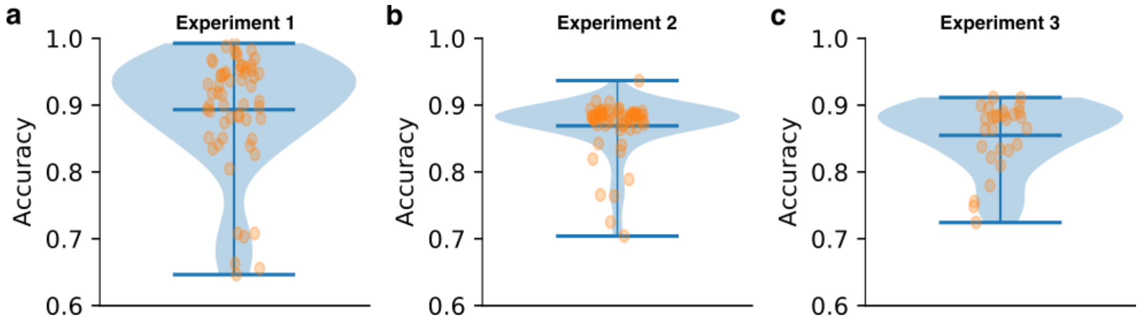

Supplementary figure S6. Accuracy in target identification. *Violin plot of observers' accuracy scores in Experiment 1* ***(a)****, 2* ***(b)****, and 3* ***(c)****. Each dot represents a single observer, and blue horizontal lines highlight the populations' means. The shaded area depicts a smoothed histogram (observer frequency indications per binned accuracy) of the population scores.*

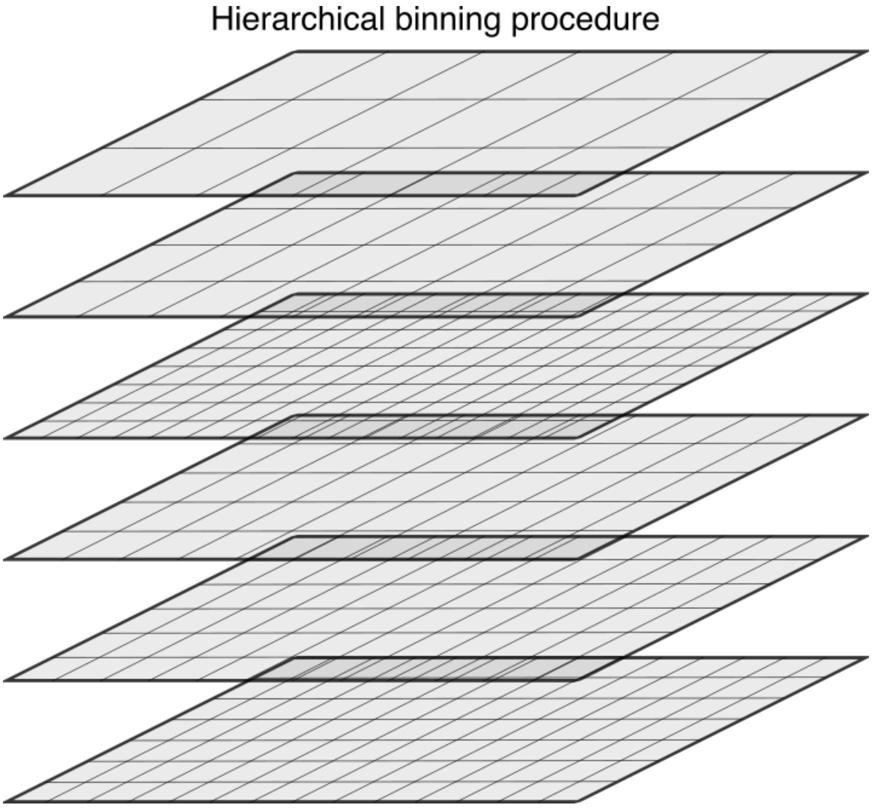

Supplementary figure S7. Hierarchical binning procedure. *The attention maps (see* ***figure 2b****,* ***3c****) reflect pupil response amplitudes averaged within bins of horizontal and vertical positions of probe centres. Multiple maps were created with different bin sizes, and a final map was the average of the multiple maps.*
